# Plasma PLTP activity is inversely associated with HDL-C levels

**DOI:** 10.1186/1743-7075-6-49

**Published:** 2009-11-30

**Authors:** Xueying Chen, Aijun Sun, Ather Mansoor, Yunzeng Zou, Junbo Ge, Jason M Lazar, Xian-Cheng Jiang

**Affiliations:** 1 Institute of Cardiology, Zhongshan Hospital, Fudan University, Shanghai, PR China; 2 Division of Cardiovascular Medicine, SUNY Downstate Medical Center, Brooklyn, NY, USA; 3 Department of Cell Biology, SUNY Downstate Medical Center, Brooklyn, NY, USA

## Abstract

Phospholipid transfer protein (PLTP) is an important modulator of lipoprotein metabolism, including interparticle phospholipid transfer, remodeling of HDL, cholesterol and phospholipid efflux from peripheral tissues, and the production of hepatic VLDL. PLTP also plays an important role in inflammation and oxidative stress. Accordingly, PLTP has been implicated in the development of atherosclerosis. In this study, we evaluated the association between PLTP activity and lipoprotein metabolism in a Chinese patients cohort with or without coronary heart disease (CHD group n = 407, control group n = 215), the PLTP activity was measured and PLTP genotyping was screened for sequence anomalies by PCR. We found that human plasma PLTP activity was negatively associated with plasma HDL and apoA-I levels, and positively associated with plasma TG, apoB and apoE levels. We also found that PLTP rs2294213 polymorphism was tended to be associated with increased plasma PLTP activity.

## 

Plasma phospholipid transfer protein (PLTP) is a lipid transfer glycoprotein that binds to and transfers a number of compounds including phospholipids, diacylglycerides, unesterified cholesterol, and lipopolysaccharidesl. In humans, PLTP activity has been shown to be positively and independently related to coronary heart disease (CHD) [1] In addition, previous studies showed that PLTP activity was increased with aging [2], obesity [3,4], type 1 and type 2 diabetes [4-6]. It is known that PLTP is involved in high-density-lipoprotein (HDL) metabolism in that human plasma PLTP activity was either positively [7,8] or negatively[2,9] correlated with HDL levels in various patient populations.

PLTP deficiency has not been found in humans while numerous PLTP polymorphisms were identified. Tahvanainen et al. reported 6 PLTP intragenic polymorphisms: c.-79G>T, c. -56G>A, c.-37T>C, c.-31A>G, p.Phe2Leu, Arg121Trp, and 2 neutral polymorphisms, however, there were no significant associations between these polymorphisms and plasma PLTP activity[2]. Similarly, Bossé and coworkers identified 2 intronic variants of the PLTP gene, 1 in intron 1 (c.-87G>A) and the other in intron 12 (c.1175+68T>G)[10]. Although both of these polymorphisms were associated with obesity-related phenotypes but there was still no significant associations between these polymorphisms and plasma PLTP activity [10]. Aouizerat and colleagues observed p.R235W mutation but not p.E72G, p.S119A, and p.S124Y in the PLTP codon region was related to decreased plasma PLTP activity and c.-34G>C allele was associated with higher HDL-C [11]. It was also reported that possession of the rs2294213 minor allele could increase HDL-C independent of triglycerides[12], but it remains unknown whether this polymorphism could influence PLTP activity and is linked with coronary heart disease(CHD) or not. In the present study, we confirmed the PLTP polymorphism (rs2294213) in a Chinese population and evaluate its relationship with plasma PLTP activity, lipid profiles and CHD.

## Methods

### Study Population

A total of 622 subjects underwent diagnostic coronary angiography for chest pain in Zhongshan Hospital between 2004 and 2005 were included in this study. Cardiovascular risk factors/diseases were obtained by clinical history. Coronary artery disease (CHD) (n = 407) was diagnosed with the presence of a stenosis ≥50% in at least 1 major coronary artery territory (left anterior descending, left circumflex or right coronary artery). Subjects who had <50% stenosis in any coronary artery served as the controls (n = 215). Exclusion criteria were evidence of significant concomitant cardiac and non-cardiac disease including severe valvular heart disease, known cardiomyopathy, malignancy, or febrile condition. Diabetes mellitus was diagnosed by clinical history, use of hypoglycemic medications or a fasting blood sugar level >7.0 mmol/L (125 mg/dL); hypertension was defined in patients receiving antihypertensive treatment or with known diagnosis of hypertension (blood pressure ≥140/90 mmHg). The study was approved by the Institutional Review Boards of Fudan University.

### Laboratory Evaluation

Subjects were advised to fast for at least 8 hours prior to blood sampling. Lipid profile was determined with Hitachi 7600 biochemistry autoanalyzer. Triglyceride (TG), total cholesterol (TC) and high-density lipoprotein-cholesterol (HDL-C) were measured with enzymatic methods (TG, Shanghai Kehuadongling Diagnostics Co., Ltd.; TC, Shanghai Kehuadongling Diagnostics Co., Ltd.; HDL-C, PEG-modified enzyme HDL-C assay, Kyowa Medex Co.). Low-density lipoprotein-cholesterol (LDL-C) was calculated according to the Friedewald formula. ApoA-I, apoE, apoB, and Lipoprotein(a) [Lp(a)] were determined by immunoturbidimetric assays (apoA-I, apo E, and apo B, DiaSyA Diagnostics; Lp(a), Nittobo Boseki Co. Ltd).

### PLTP Genotyping

PLTP genotype was identified for each subject by genomic DNA PCR using sense primer TAAAGGCGGCTGGAACAACCCTG-3' and anti-sense primer GCGTTCTCCTTCATCGGCTCT-3'. The SNP reference number was identified by placing the flanking sequences with 30 base paires from each side of the SNP into the NCBI SNP database to do BLAST analysis. The polymorphism (rs2294213) is located on PLTP gene Intron 1.

### PLTP Activity Assay

PLTP activity was measured with an assay kit (Cardiovascular Targets Inc., New York, NY, USA). The kit includes both donor and acceptor particles. Incubation of donor and acceptor particles with 3 μl of human plasma results in PLTP-mediated transfer of fluorescent phospholipid, which is present in a self-quenched state when associated with the donor. The transfer is determined by the increase in fluorescence intensity as the fluorescent lipid is removed from the donor and transferred to the acceptor. The interassay coefficient of variation of the PLTP activity was 3.3 ± 0.5%. The linear range of PLTP activity in this assay was between 1 and 7 μl of plasma. Three freeze-thaw cycles of plasma did not influence the assay. To validate the novel PLTP activity assay, we compared the results with those obtained by the classic radiolabeled method [13,14] There was a high degree of correlation between the 2 methods (*r *= 0.90, *p *< 0.01; *n *= 30). Laboratory personnel were unaware of the study individual assignation.

### Statistical Methods

The study subjects were grouped according to their PLTP genotype. Continuous variables were reported as means ± standard deviation (SD) and univariate analysis of variance tests were used to test statistical significance. The Pearson's X^2 ^test statistic was used to assess statistically significant differences in proportions for dichotomous and categorical variables. The distribution of PLTP activity and Lp(a) levels was positively skewed and so were naturally log transformed prior to analyses. Multivariate analysis of variance was performed to evaluate the relationship between PLTP genotype and PLTP activity level. The dependent variable was log transformed PLTP activity level. The independent variables included PLTP genotype, gender, and presence/absence of diabetes, hypertension, smoking, low HDL and CHD. Age and BMI were entered into the model as covariates. Selected interaction effects were tested between the independent variables in the model. Estimated geometric means of PLTP activity level were generated for each independent variable using reverse-log transformation of the ANOVA generated marginal means. LSD test was performed for multiple comparisons. To further investigate the relationship between PLTP activity and plasma lipid parameters, univariate and multivariate linear regression analyses were performed and estimated expected change in PLTP activity was determined for a unit change in each lipid parameter. All analyses were done using SPSS version 17 analytical software (SPSS Inc., Chicago, IL).

## Results

Based on genomic DNA PCR and sequence, we detected the rs2294213 PLTP polymorphism which is located on PLTP gene Intron 1. Of the 622 subjects, 277 (44.5%) had 'GG' polymorphism PLTP genotype, 276 (44.4%) had 'CG' PLTP genotype and 69 (11.1%) had 'CC' polymorphism. Obtained genotype frequencies were compared to the calculated frequencies based on the Hardy-Weinberg equilibrium and no significant differences were observed (χ^2 ^= 0.000631, P = 0.980 for all participants; χ^2 ^= 1.443, P = 0.230 for the control group; χ^2 ^= 0.708, P = 0.400 for the CHD patient group). The clinical characteristics of studied patients are shown in Table 1. Age, gender, BMI, and lipid profiles were similar among groups. Prevalence of clinical risk factors including diabetes, hypertension, smoking, low HDL and CHD were also similar among the 3 groups. Plasma PLTP activity was significantly higher in subjects with 'CC' polymorphism than that in subjects with 'GG' and 'GC' polymorphisms (26.7 ± 12.8 pmol/μL/hr vs. 23.7 ± 14.7/24.7 ± 14.0 pmol/μL/hr; p = 0.04). Testing with an LSD test, subjects with 'CC' polymorphism had significantly higher PLTP activity as compared to those with 'GG' one (P = 0.02). However, no statistical significant difference of PLTP activity was found between the 'CC' and 'GC' groups (P = 0.26). The similar result was found between the 'GG' and 'GC' groups (P = 0.07).

**Table 1:** Patient Characteristics.

Characteristics†	PLTP* genotype (N)			p-value‡
	**gg (277)**	**gc(276)**	**cc(69)**	

Age (years)	61.3 ± 10.5	63.3 ± 9.9	63.0 ± 9.4	0.06
Gender				
Men (%)	211(76.2)	222(80.4)	54(78.3)	0.64
Women (%)	65(23.5)	54(19.6)	15(21.7)	
BMI^§ ^(kg/m^2)^	24.7 ± 2.9	24.5 ± 2.9	24.5 ± 3.2	0.55
Diabetes (%)	56(20.21)	51(18.5)	11(15.9)	0.69
Hypertension (%)	171(61.7)	175(63.4)	23(33.3)	0.77
Smoking (%)	126(45.5)	133(48.2)	29(42.0)	0.61
CHD^|| ^(%)	176(63.5)	189(68.4)	42(60.8)	0.33
**PLTP activity (pmol/μl/h)^**€**^**	**23.7 ± 14.7**	**24.7 ± 14.0**	**26.7 ± 12.8**	**0.04** **0.02^†† ^(cc vs gg)** **0.26 ^**†† **^(cc vs gc)** **0.07 ^†† ^(gg vs gc)**
Total-C^¶ ^(mmol/L)	4.71 ± 0.96	4.41 ± 1.03	4.40 ± 0.99	0.74
TG^# ^(mmol/L)	1.74 ± 0.87	1.71 ± 1.08	1.8 ± 0.99	0.79
LDL-C (mmol/L)	2.65 ± 0.85	2.60 ± 0.88	2.55 ± 0.89	0.67
HDL-C (mmol/L)	1.03 ± 0.26	1.04 ± 0.29	1.01 ± 0.27	0.94
Low HDL-C (<0.8 mmol/L) (%)	81(29.2)	80(29.0)	21(30.4)	0.94
LDL-C/HDL-C	2.74 ± 1.28	2.63 ± 0.99	2.71 ± 1.24	0.50
ApoA-I** (g/L)	1.07 ± 0.19	1.06 ± 0.21	1.03 ± 0.21	0.18
apoB (g/L)	0.82 ± 0.42	0.82 ± 0.42	0.79 ± 0.26	0.87
apoE (mg/L)	47.99 ± 25.57	47.34 ± 18.39	45.17 ± 13.00	0.63
Lp(a)^€ ^(mg/L)	179.76 ± 157.41	202.89 ± 187.61	199.51 ± 227.73	0.30

* PLTP = Phospholipid transfer protein; † Means are presented ± standard deviation; ‡ p-values for differences in means were calculated using t test statistics, p-values for differences in proportions were calculated using Pearson's X^2 ^test, †† p-values for differences using an LSD test for multiple comparisons; § BMI = body mass index; || CHD = Coronary Heart Disease with ≥50% coronary artery stenosis; € compared after log_10 _transformation; ¶ Cholesterol; # Triglycerides; ** Apolipoprotein.

As shown in Table 2, based on univariate analyses, plasma PLTP activity was significantly associated with male gender, presence of low HDL and with PLTP genotype. In multivariable-adjusted analysis, the effect of gender was attenuated though low HDL and PLTP genotype remained independently associated with PLTP activity.

**Table 2:** Univariate and multivariate estimates of PLTP activity levels.

Variable	Unadjusted estimated PLTP^† ^activity (Mean ± SE)	p-value	Adjusted* estimated PLTP^† ^activity (Mean ± SE)	p-value
**Gender**				
Male	21.20 ± 1.03	0.03	24.15 ± 1.06	0.52
Female	18.27 ± 1.06		23.01 ± 1.08	
**Diabetes**				
Present	22.66 ± 1.06	0.08	24.38 ± 1.07	0.35
Absent	20.05 ± 1.03		22.80 ± 1.05	
**Hypertension**				
Present	20.13 ± 1.04	0.36	22.86 ± 1.05	0.27
Absent	21.21 ± 1.05		24.38 ± 1.06	
**Smoking**				
Smoker	21.58 ± 1.04	0.09	24.04 ± 1.07	0.52
Nonsmoker	21.58 ± 1.04		23.12 ± 1.05	
**CHD**^**‡**^				
Present	20.70 ± 1.03	0.68	23.50 ± 1.05	0.93
Absent	20.21 ± 1.05		23.66 ± 1.07	
**Low HDL-C**^**§**^				
Present	27.20 ± 1.05	<0.01	28.18 ± 1.06	<0.01
Absent	18.46 ± 1.03		19.77 ± 1.05	
**PLTP Polymorphism Type**				
gg	19.41 ± 1.04	0.03	21.18 ± 1.05	0.03
cg	21.26 ± 1.04		23.50 ± 1.06	
cc	23.59 ± 1.09		26.36 ± 1.09	

* Covariates appearing in the multivariate model were evaluated at values of age = 62.43 years and BMI = 25.21 kg/m^2^; † PLTP= Phospholipid transfer protein; ‡ CHD = Coronary Heart Disease with ≥50% coronary artery stenosis; § Low HDL-C defined as HDL-cholesterol level <0.8 mmol/L.

We next sought to evaluate PLTP activity and its relation with plasma lipid levels. In univariate analysis, PLTP activity was significantly positively associated with plasma triglyceride (TG), apolipoprotein (apo)B and apoE, while it was negatively associated with plasma HDL and apoA-I. The effect of plasma TG was attenuated on multivariable-adjusted analysis (Table 3).

**Table 3:** Univariate and multivariate plasma lipid correlates of PLTP activity

Predictor variables	Unadjusted expected change in PLTP activity (Units)	p-value	Adjusted† expected change in PLTP† activity (Units)	p-value
Total-Cholesterol, per mmol/L	-1.01	0.06	-1.01	0.05
Triglycerides, per mmol/L	1.03	0.01	-1.00	0.92
HDL-Cholesterol, per mmol/L	-1.15	<0.01	1.27	<0.01
LDL-Cholesterol, per mmol/L	-1.01	0.35	-1.01	0.18
apoA-I, per g/L	-1.48	<0.01	-1.75	<0.01
apoB, per g/L	1.09	<0.01	1.10	<0.01
apoE, per mg/L	1.00	<0.01	1.00	<0.01
Lp(a), per mg/L	1.03	0.29	1.05	0.11

PLTP = Phospholipid transfer protein; † Adjusted for age, gender, BMI, PLTP Polymorphism, and presence or absence of significant CHD.

## Discussion

The major finding of our study is that PLTP activity inversely associated with plasma HDL-C levels. Importantly, the significant relation between PLTP activity and HDL-C persisted even after adjustment for age, gender, BMI and presence or absence of significant CHD.

There is growing evidence that the biological role(s) of PLTP is related to HDL metabolism. Transgenic mice that overexpressing human PLTP at high levels were previously been generated. Compared with WT mice, these mice show a 2.5-4.5-fold increase in PLTP activity in plasma [15]. The overexpression of PLTP resulted in a significant decrease of plasma levels of HDL-C[15], increase the formation of preβ-HDL[15], increase catabolism HDL[16,17], increased atherosclerosis[18], compared with plasma from WT mice. We created PLTP gene knockout (KO) mice[19] which showed a marked decrease in HDL-C, phospholipid, and apoA-I. Furthermore, the HDL of the PLTP KO mice was enriched with protein and poor in phosphatidylcholine, and turnover studies showed a 4-fold increase in the catabolism of HDL protein and CE compared with that of WT mice [20,21]. Interestingly, both PLTP transgenic and KO mice showed the same phenotype, in terms of HDL levels. These studies could not provide a prediction for the relationship between PLTP activity and HDL levels in humans. Thus far the results from human studies are controversial. Human plasma PLTP activity and HDL levels have been shown to correlate either positively or negatively in different populations [2,7-9]. In the present study, we chose a relatively large (n = 622) and relatively uniform cohort and found that, indeed, PLTP activity is inversely related with HDL levels. Why low-HDL subjects with higher PLTP activity? It is known that in addition to promoting transfer of phospholipids from VLDL and chylomicron into HDL[19], PLTP may contribute to the remodelling of HDL particles. PLTP activity contributes to the generation of large HDL as well as small HDL particles [22,23] and may promote catabolism of these particles, thus decreasing HDL levels. Besides PLTP, there is cholesteryl ester transfer protein (CETP) in human plasma. It is well known that CETP activity is inversely related with HDL[24]. Although PLTP has no CE transfer activity, it can promote CETP activity [25]. We also reported that PLTP KO/CETP transgenic mice have significantly less CETP activity than that in CETP transgenic mice[26]. High PLTP activity might promote CETP activity, thus decreasing HDL levels in human plasma. It is known that PLTP function extends far beyond phospholipid transfer and exchange among lipoproteins. In mice, PLTP deficiency has been associated with a reduction in systemic inflammation [27,28]. Furthermore, in mouse models, PLTP deficiency has been associated with increased antioxidation potential[29], while the opposite has been observed in PLTP overexpression [30]. In addition, PLTP is also involved in ABCA1-dependent cholesterol efflux, a process which is critical for reverse cholesterol transport [31,32]. Finally, PLTP activity is closely related to obesity, diabetes mellitus, insulin resistance [3-6], left ventricular systolic dysfunction [33], and intima-media thickness[34]. Although the mechanism of these associations remains poorly defined, it is likely related to the putative role of PLTP in the development of atherosclerosis, a common underlying factor in each of these disease states.

In this study, we also found PLTP activity is positively related to plasma apoB levels (Table 3). We and other researchers have reported previously that PLTP deficiency in mice results in markedly decreased in apoB-containing lipoproteins in the circulation[35] and PLTP overexpression stimulation of VLDL secretion[36]. This effect may well have an impact on atherogenesis.

In addition, we found a PLTP polymorphism (rs2294213) has impact on PLTP activity. Plasma PLTP activity was higher in subjects with 'CC' polymorphism than that in subjects with 'GG' and 'GC' polymorphisms (26.7 ± 12.8 pmol/μL/hr vs. 23.7 ± 14.7/24.7 ± 14.0 pmol/μL/hr; p = 0.04). However, LSD test showed that except 'CC' vs 'GG' groups, there was no statistical significant difference between the 'CC' and 'GC' or 'GG' and 'GC' groups. Since there were no statistically significant associations between other polymorphisms and plasma PLTP activity [2,10], the effect of rs2294213 polymorphism on PLTP activity might be marginal.

Engler et al[12] reported that, in non-Hispanic white sample of subjects, allele frequency of rs2294213 accounted for 2.2%, 4.3%, and 7.0% in hypoalphalipoproteinemia, controls, and hyperalphalipoproteinemia groups, respectively, and this polymorphism is responsible for the increasing of HDL-C. However, the allele frequency in our study (33%) is much higher than theirs [12] and we did not observe the effect of this polymorphism on HDL-C levels. The different study population might explain the different allele frequency and different influences towards lipid levels. The relative frequency of the CC polymorphism and its relation to HDL levels merits further study.

We have reported PLTP activity is an independent risk factor for CHD[1], but we do not find the association between PLTP activity and CHD in this Chinese population, which is inconsistent with our precious study in a well-controlled and big size population [1]. This might be related to the relatively small number of patients with normal or non-obstructive CHD in our study population.

## Conclusion

Human plasma PLTP activity was inversely associated with HDL levels and positively related to plasma apoB levels in this Chinese population. Although our findings need to be confirmed in additional studies, they hold the promise that PLTP might be therapeutic target for the treatment of atherosclerosis.

## Competing interests

The authors declare that they have no competing interests.

## Authors' contributions

XC and AS participated in the design of the study, and carried out the molecular genetic studies and PLTP activity analysis. AM and JML performed the statistical analysis. YZ and JG participated in the design of the study. XCJ participated in the design of the study and drafted the manuscript. All authors read and approved the final manuscript.
